# Prediction of Coronary Artery Spasm in Patients Without Obstructive Coronary Artery Disease Using a Comprehensive Clinical, Laboratory and Echocardiographic Risk Score

**DOI:** 10.3390/jcm14248721

**Published:** 2025-12-09

**Authors:** Yu-Ching Lee, Ian Y. Chen, Ming-Jui Hung, Chi-Tai Yeh, Nicholas G. Kounis, Patrick Hu, Ming-Yow Hung

**Affiliations:** 1 Graduate Institute of Athletics and Coaching Science, National Taiwan Sport University, No. 250 Wenhua 1st Rd., Guishan, Taoyuan 33301, Taiwan; b8404056@tmu.edu.tw; 2 Biostatistics Research Center, Taipei Medical University, Taipei City 11031, Taiwan; 3 Division of Cardiovascular Medicine, Department of Medicine, Department of Radiology, Stanford Cardiovascular Institute, Stanford University School of Medicine, Stanford, CA 94305, USA; iychen@stanford.edu; 4 Cardiology Section, Medical Service, Veterans Affairs Palo Alto Health Care System, Palo Alto, CA 94304, USA; 5 Division of Cardiology, Department of Medicine, Chang Gung Memorial Hospital, Keelung, Chang Gung University College of Medicine, Keelung 20420, Taiwan; hmj1447@cgmh.org.tw; 6 Department of Medical Research and Education, Shuang Ho Hospital, Taipei Medical University, New Taipei City 23561, Taiwan; ctyeh@s.tmu.edu.tw; 7 Department of Medical Laboratory Science and Biotechnology, Yuanpei University of Medical Technology, Hsinchu 300102, Taiwan; 8 Department of Cardiology, University of Patras Medical School, 26221 Patras, Greece; ngkounis@otenet.gr; 9 Department of Internal Medicine, School of Medicine, University of California, Riverside, CA 92521, USA; dr.hu.md@gmail.com; 10 Department of Cardiology, Riverside Medical Clinic, Riverside, CA 92506, USA; 11 Division of Cardiology, Department of Internal Medicine, Shuang Ho Hospital, Taipei Medical University, New Taipei City 23561, Taiwan; 12 Division of Cardiology, Department of Internal Medicine, School of Medicine, College of Medicine, Taipei Medical University, Taipei City 11031, Taiwan; 13 Taipei Heart Institute, Taipei Medical University, Taipei City 110301, Taiwan

**Keywords:** coronary artery spasm, echocardiography, risk prediction score

## Abstract

**Background:** The lack of an accurate coronary artery spasm (CAS) risk prediction model highlights the failure to consider dynamic coronary health and reveals a gap in understanding CAS. **Methods:** A total of 913 Taiwanese patients (460 women and 453 men) with suspected ischemic heart disease but without angiographic obstructive coronary artery disease were subjected to intracoronary methylergonovine testing during the period 2008–2025. **Results:** The study included 645 CAS cases (70.6%) and 268 non-CAS controls (29.4%). The multivariable logistic regression model identified 10 variables significantly associated with CAS (*p* < 0.05): male sex, smoking, low systolic and diastolic blood pressure, reduced B-type natriuretic peptide levels, elevated low-density lipoprotein levels, increased relative wall thickness at end-systole, high left ventricular mass index, low e’(l) values, and high Tei index. Discrimination performance was moderate, with an AUC value of 73.8% that dropped to 72.4% after bootstrapped internal validation, suggesting the potential generalizability of the derived model. The total score ranged from 36 to 98, representing a predicted probability between 12% and 98%, respectively. **Conclusions:** While a total score of ≥58 with the probability of CAS exceeding 50% indicates a significant chance of undiagnosed CAS, for patients with a total score ≥ 69 and a high probability of CAS ≥ 75%, coronary catheterization with CAS provocation testing is strongly recommended for a definite diagnosis. The simple 10-variable scoring model allows ranking of at-risk populations and is designed to be used as a screening tool rather than a diagnostic adjunct, enabling more efficient diagnostic resource allocation.

## 1. Introduction

Epicardial large coronary artery spasm (CAS) is a severe narrowing of coronary arteries due to intense vasoconstriction, leading to angina, myocardial ischemia, infarction, or sudden cardiac death [1,2]. Unlike obstructive coronary artery disease (CAD), CAS is linked to risk factors such as smoking, age, and elevated C-reactive protein [3], but not to diabetes or hypertension [3], highlighting key pathophysiological differences between CAS and CAD. Traditional CAD risk models, including the Framingham Heart Study [4], often do not account for key factors such as imaging data, which can lead to an oversimplified view of dynamic coronary health and overlook important predictors for CAS. We have demonstrated that CAS patients have a high risk of stroke and cardiovascular mortality [5]. Therefore, while invasive provocation testing remains the gold standard for CAS diagnosis, there is a need for effective non-invasive scoring systems for earlier identification of CAS and guiding medical therapy. To date, only one clinical score system with good accuracy in predicting CAS has shown an area under the curve of 0.952 [6]. However, its variables—such as asthma (<4% prevalence among CAS patients) [7], varied ECG findings [3], and the moderate sensitivity of hyperventilation testing in patients with less frequent CAS attacks [3]—highlight the need for more reliable CAS risk prediction models.

Multiple risk factors may coexist and interact to increase the likelihood of developing CAS [3], underscoring the need for a multifactorial approach to prevention. CAS risk estimation typically incorporates factors such as age, sex, medical history, smoking exposure, C-reactive protein levels, symptoms, and ischemic electrocardiographic changes during attacks [3]. In addition, because B-type natriuretic peptide (BNP), secreted by ventricular cardiomyocytes, has potent vasodilatory effects predominantly on epicardial conductance vessels, its deficiency may contribute to the development of epicardial CAS [8]. Gohbara et al. have demonstrated that BNP, a potent vasodilator, is lower in CAS-induced than CAD-induced non-progressive ST-elevation acute coronary syndrome [9]. Therefore, we hypothesized that BNP could differentiate between CAS-related and non-CAS-related angina in patients without obstructive CAD. In addition, abnormal left ventricular (LV) filling and relaxation are early, reversible indicators of myocardial ischemia [10]. Therefore, quantitative echocardiography should effectively distinguish between CAS-induced and non-CAS-induced angina. Notably, in the assessment of global systolic and diastolic function, the Tei index is a simple Doppler parameter independent of heart rate and blood pressure, applies to left and right ventricular systolic and diastolic dysfunction, does not rely on geometric assumptions and is highly reproducible [11]. Endothelium-dependent vasodilation is progressively impaired with abnormal LV geometry [12], and remote tissue can be adversely affected by inflammation from ischemic sites, as evidenced by hypertrophy in the non-inflamed posterior wall following anterior wall myocardial infarction in mice [13]. Relative wall thickness (RWT), derived from posterior wall thickness, has been identified as a cardiovascular risk factor [14], and LV geometric patterns classified by RWT provide additional predictive value for cardiovascular events [15]. However, the association between CAS and abnormal LV remodeling or relaxation remains poorly understood.

Accurate prediction and early diagnosis of CAS are crucial for preventing cardiovascular events, especially in individuals with multiple mild abnormalities. While CAS subjects rarely present with more than four risk factors, no clinical diagnostic score incorporating echocardiographic parameters has been developed for CAS. Therefore, we assessed how well a risk score combining clinical data, blood tests, and echocardiographic measures can identify undiagnosed CAS without obstructive CAD in primary care.

## 2. Materials and Methods

### 2.1. Study Population

This was a retrospective analysis of a prospective cohort enrolled from November 2008 to March 2025. Throughout the study period, all participants received care from the same physician, Dr. Ming-Yow Hung, both in the clinic and the hospital.to ensure a high level of data completeness, consistent clinical decision-making and minimized variability in treatment practices across the cohort. During this period, all 913 consecutive patients with suspected ischemic heart disease but without angiographic evidence of obstructive CAD were subjected to intracoronary methylergonovine testing at Shuang Ho Hospital, Taipei Medical University. Patients were grouped according to the presence or absence of CAS. Diagnostic criteria for CAS included spontaneous chest pain at rest; ST-segment elevation or depression on electrocardiogram that was relieved by sublingual administration of nitroglycerin; and a positive result on intracoronary methylergonovine provocation testing. The control group included patients with atypical, non-exertional chest pain and negative intracoronary methylergonovine provocation testing. The exclusion criteria included obstructive CAD, previous coronary angioplasty or myocardial infarction, coronary microvascular spasm, severe valvular heart disease, inflammatory manifestations probably associated with noncardiac diseases (e.g., infections and autoimmune disorders), liver disease/renal failure (serum creatinine level > 2.5 mg/dL), collagen disease, malignancy and missing blood samples (complete-case analysis). None of our patients had allergic or hypersensitivity conditions. This study was approved by the Taipei Medical University-Joint Institutional Review Board (approval number: 201011004), and all patients gave written informed consent.

### 2.2. Clinical Data

Patients were assessed for the presence of the following cardiac risk factors: age, sex, cigarette smoking status, diabetes mellitus, hypercholesterolemia and hypertension. Current smoking status was defined as at least 0.5 pack-year and having smoked at least 1 cigarette within 3 weeks of cardiac catheterization. Patients were determined to have diabetes mellitus if they were currently on dietary treatment and/or medical therapy for diabetes mellitus. The average baseline self-measured systolic and diastolic seated blood pressure at home was the mean of a minimum of 12 readings total optimally for 7 days, with a minimum of 3 days. Hypertension was defined as blood pressure of >130/80 mm Hg or receiving antihypertensive treatment. Hypercholesterolemia was diagnosed in patients with serum total cholesterol > 200 mg/dL. All patients underwent echocardiography, when heart rates were recorded, before coronary angiography and within 2 weeks of the last angina. The blood pressure levels were measured at the time of coronary angiography.

### 2.3. Laboratory Analysis

Data for serum creatinine, estimated glomerular filtration rate, hemoglobin, hematocrit, platelet counts, white blood cell count, monocyte counts, BNP, high sensitivity C-reactive protein, blood glucose, hemoglobin A1c, total cholesterol, triglycerides, high-density lipoprotein and low-density lipoprotein were obtained on admission within 24 h prior to angiography.

### 2.4. Coronary Angiography and Intracoronary Methylergonovine Testing

Coronary angiography was performed within 2 months of chest pain using the standard Judkins technique via a femoral or radial approach. Nitrates and calcium antagonists were withdrawn for ≥24 h before coronary angiography [2]. The LV ejection fraction was calculated using Simpson’s method. Selective left and right coronary angiography were performed in multiple axial and hemiaxial projections. Obstructive CAD was defined as ≥50% diameter reduction in lumen caliber after administration of intracoronary nitroglycerin (100 μg) [16]. Intracoronary methylergonovine (Methergin^®^; Novartis, Basel, Switzerland) provocation testing was performed in succession if no obstructive CAD was found. Methylergonovine was administered stepwise (1, 5, 10, and 30 μg) first into the right coronary artery and subsequently into the left coronary artery. Provocation testing for CAS was considered positive when there was a >70% reduction in luminal diameter compared to post-intracoronary nitroglycerin and when there was associated angina and/or ST depression or elevation [17]. Provocation testing was stopped with intracoronary administration of 100–200 μg nitroglycerin (Millisrol^®^; G. Pohl-Boskamp, Hohenlockstedt, Germany). The observation of reversal changes in the coronary artery diameter further confirmed the diagnosis of CAS. Spontaneous CAS was defined as the relief of >70% diameter stenosis after intracoronary administration of 100–200 μg nitroglycerin.

### 2.5. Echocardiographic Methods

Echocardiograms were obtained using a Philips iE33 (Philips Ultrasound, Bothell, WA, USA), with the patients breathing quietly in the left decubitus position. The patients underwent 2-dimensional and M-mode echocardiographic examinations. Left atrial antero-posterior and LV diameters, wall thickness and ejection fraction were measured according to the guidelines of the American Society of Echocardiography [18,19]. The LV ejection fraction was calculated according to modified Simpson’s rules [20]. End-diastolic LV dimensions were used to calculate LV mass using an anatomically validated formula [21]. Left atrial (LA) diameter was measured in the parasternal long-axis view from the trailing edge of the posterior aortic/anterior LA complex at end-systole as the anteroposterior linear diameter, using 2-dimensional echocardiographic guidance to position the cursor as recommended [22]. RWT at end-diastole (RWTLVEDD) was calculated as posterior wall thickness/internal radius [23]. Increased RWTLVEDD was considered present when the ratio was ≥0.43 [24]. RWT at end-systole (RWTLVESD) was also calculated by a similar formula because this contributes to the LV geometric component of passive ventricular stiffness that is superimposed on the active relaxation in early diastole [14]. LV hypertrophy was considered present when the LV mass index (LVMI) for body surface area was >116 g/m^2^ for men and >104 g/m^2^ for women [25]. The combinations of LVMI and RWTLVEDD defined 4 LV geometric patterns: normal geometry, concentric hypertrophy with increased RWTLVEDD and LV hypertrophy, eccentric hypertrophy with normal RWTLVEDD and LV hypertrophy, and concentric remodeling with increased RWTLVEDD and normal LVMI [26].

Peak velocities of early diastolic filling (E), late diastolic filling (A) and deceleration time were derived from transmitral Doppler recordings. Isovolumic relaxation time (IVRT) was measured at the apical 5-chamber view with the sampling volume positioned between the mitral valve and the LV outflow tract as the time taken from the closure of the aortic valve to the opening of the mitral valve. Doppler tissue echocardiography was performed at transducer frequencies of 3.5–4.0 MHz, adjusting the spectral pulsed Doppler signal filters until a Nyquist limit of 15–20 cm/s was reached, and using the minimum optimal gain. The monitor sweep speed was set at 50–100 mm/s to optimize the spectral display of myocardial velocities. Tissue Doppler-derived early diastolic velocities (e’) and late diastolic velocities (a’) were derived from the lateral and medial mitral annulus. The lateral and mitral E/e’ ratio was subsequently calculated. While τ, the time constant of the LV pressure decay at isovolumic relaxation, is a sensitive index of myocardial ischemia, τ with a zero asymptote assumption (τ_0_) was estimated using the following equation: τ_0_(ms) = IVRT_doppler_/[ln(P_s_) − ln(P_PCWP_)] [27], where P_s_ is the systolic blood pressure and PCWP is the mean pulmonary capillary wedge pressure. All procedures complied with the guidelines from the American Society of Echocardiography [28].

### 2.6. Statistical Analysis

Baseline characteristics between the CAS and control groups were compared using the chi-square test for categorical variables, the independent sample *t*-test for continuous variables, and the Mann–Whitney U-test for BNP and high-sensitivity C-reactive protein due to their non-normal distribution. The potential predictive factors comprised 52 parameters, including 8 demographic variables (e.g., age, sex, and smoking status), 18 vital signs and laboratory results (e.g., systolic blood pressure and creatinine), and 26 echocardiographic features, as detailed in Table 1. In the first step, univariate logistic regression analyses were conducted to identify potential factors associated with CAS risk based on all baseline characteristics. Variables with a significance level of less than 0.15 in the univariate analyses were then included in a multivariable logistic regression model with backward elimination. Discriminative performance was evaluated using the area under the curve, with the value above 0.7 considered acceptable.

The performance of calibration was evaluated by comparing the predicted probability to the actual observed probability across the deciles of the predicted probabilities. Calibration performance was assessed by comparing predicted probabilities to the actual observed probabilities across deciles of the predicted values, supplemented by the Hosmer–Lemeshow goodness-of-fit test. The generalizability of the obtained model was assessed through internal validation using 1000 bootstrap samples, providing reasonably valid estimates of expected optimism [29]. At last, the results of the final multivariable logistic regression model were converted into a simplified point system to enhance clinical applicability. The key concept involves rounding off regression coefficients, with further details available in a previous report [30]. All tests were 2-tailed, and *p* < 0.05 was considered statistically significant. Data analyses were conducted using R, version 4.2.2 (R Foundation for Statistical Computing) with package ‘rms’ (Frank E. Harrell Jr.).

## 3. Results

### 3.1. Patient Characteristics

A total of 913 patients with complete baseline data were included in the study, comprising 645 (70.6%) CAS cases and 268 (29.4%) non-CAS controls. Table 1 presents detailed baseline characteristic data. The mean age was 57.1 years in the CAS group and 54.8 years in the control group, with a significant difference (*p* = 0.014). The proportion of male participants was higher in the CAS group (54.6% vs. 37.7%, *p* < 0.001). Regarding other demographics, vital signs and laboratory results, patients with CAS had a larger body surface area; were more likely to smoke; had lower systolic and diastolic blood pressure levels; and had higher hemoglobin and hematocrit levels, lower B-type natriuretic peptide (BNP) levels, elevated triglycerides and low-density lipoprotein (LDL) levels, and lower high-density lipoprotein levels (*p* < 0.05). In CAS, single-vessel spasm was the predominant observation (91%), with the right coronary artery being the most frequently affected (81%). Several echocardiography parameters differed significantly between groups, as detailed in Table 1.

### 3.2. Associated Factors of CAS

Table 2 presents the univariate logistic regression analysis for the preliminary screening of potential factors associated with CAS.

The final multivariable logistic regression model identified male sex, smoking, lower systolic and diastolic blood pressure, lower BNP levels, higher LDL levels, greater RWTLVESD, larger LVMI, lower e’(l) values and a higher Tei index as significant factors associated with CAS (*p* < 0.05; Table 3). The variance inflation factors for the 10 variables ranged from 1.04 to 1.54, indicating that the final multivariable model did not exhibit concerns regarding multicollinearity.

**Figure 1:**
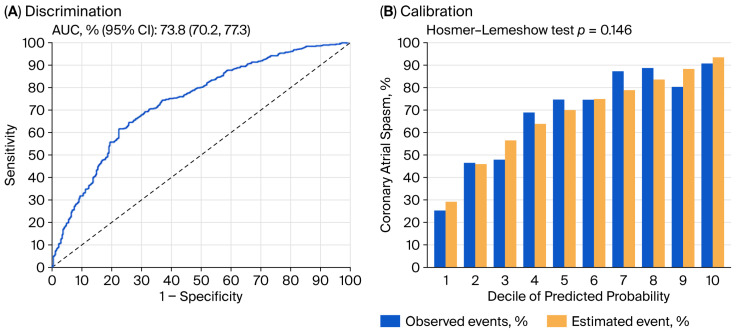
Discrimination (**A**) and calibration (**B**) performance of derived multivariable logistic regression model for predicting coronary artery spasm. AUC: area under the curve; CI: confidence interval.

The model’s discrimination performance was modest, with an area under the curve value of 73.8% (Figure 1A). After correcting for optimism through bootstrapped internal validation, the area under the curve slightly decreased to 72.4%, suggesting the potential generalizability of the derived model. The model also demonstrated good calibration, with non-substantial discrepancy (*p*-value of Hosmer–Lemeshow test = 0.146) between the predicted and actual probabilities (Figure 1B).

### 3.3. Clinical Use of the Prediction Model

Table 4 presents the simplified scoring function for CAS prediction, derived from the multivariable logistic regression model. For continuous variables, we categorized them using relatively fine intervals whenever possible—for example, blood pressure was grouped in 10-mmHg increments, and BNP was categorized in 50 pg/mL increments.

**Figure 2:**
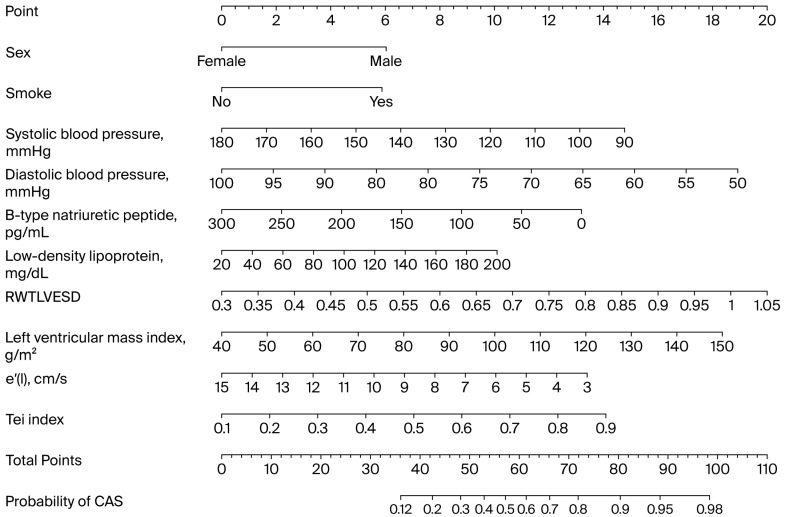
Nomogram illustrating relationships between predictive variables and probability of coronary artery spasm, derived from logistic regression model. CAS: coronary artery spasm; LVESD: left ventricular end-systolic diameter.

The total score ranged from 36 to 98, corresponding to a predicted CAS probability between 12% and 98%, respectively. While a simplified scoring function is easy to calculate manually, a nomogram offers greater flexibility and visual clarity, accommodating continuous variables and nonlinearities for better individualized risk assessment and clinical decisions (Figure 2).

## 4. Discussion

We found that, for individuals without obstructive CAD, a simple comprehensive 10-variable scoring model (sex, smoking status, SBP, DBP, BNP, LDL, LVMI, RWTLVESD, e’(l) and Tei) based on a combination of clinical, echocardiographic data and blood tests could offer a non-invasive means of identifying patients at earlier stages of developing CAS. The probability of CAS was <25% when the total score was ≤45; hence, it was reasonable to follow up with these patients without performing coronary catheterization. A total score of ≥58 with the probability of CAS exceeding 50% indicates a significant chance of CAS being present, and specific non-invasive provocative hyperventilation testing should be considered. For patients with a total score ≥ 69 and a high probability of CAS ≥ 75%, coronary catheterization with CAS provocation testing is strongly recommended for a definite diagnosis. Our well-calibrated CAS risk score model had good discrimination, which could help prioritize risks to effectively allocate diagnostic coronary catheterization and guide treatments.

Two CAS diagnostic scores [6,9] and one Japanese Coronary Spasm Association prognostic score in CAS patients [31] have been developed. The clinical diagnostic score proposed by Lin et al. [6] assigns points to six factors—angina at rest, positive hyperventilation test, allergies, asthma, ST-segment elevation, and myocardial bridge—to predict CAS, with scores of 11–12 indicating high diagnostic accuracy. Notably, Lin et al.’s score emphasizes hyperventilation testing (high specificity 100%, moderate sensitivity 62%), risking missed cases of true CAS. The other diagnostic score differentiates CAD from CAS in patients with acute coronary syndrome but lacks validation for stable angina. Moreover, it should be noted that a positive result on spasm provocation testing performed soon after acute coronary events may not be equivalent to a definitive diagnosis of CAS. Both studies suffer from methodological issues such as random data splits and imbalanced datasets: basic random train-validation data splits can lead to noisy performance estimates and reduce the data available for training, especially with small datasets; random splits disregard the temporal dependency in time series data, leading to unrealistic evaluation; random splits might not maintain the class distribution in imbalanced datasets, potentially leading to biased training or validation sets; information from the validation set can inadvertently “leak” into the training process, leading to overly optimistic performance estimates. Nevertheless, our observation that patients with CAS exhibited low BNP levels is consistent with their findings. The Japanese Coronary Spasm Association score predicts adverse events in CAS patients but is prognostic, not a screening tool.

Men are more likely than women to develop epicardial CAS in both East Asian and Western populations [2,3], partly due to higher rates of cigarette smoking [32]. Middle-aged women tend to have greater resting coronary flow than men [33] possibly linked to cardiac autonomic differences [34], which may lower their coronary flow reserve [35,36]. For similar degrees of epicardial stenosis, women may show higher fractional flow reserve because of sex-based vasomotion differences [37], though microvascular resistance remains comparable between sexes with ischemia and nonobstructive CAD [35]. Factors such as increased smoking, reduced resting coronary flow, lack of estradiol-mediated vasodilation, and decreased fractional flow reserve may explain the higher CAS prevalence in men. Smoking promotes CAS via C-reactive protein-induced IL-6 expression, nicotine activation of α7-nicotinic acetylcholine receptors, adrenergic stimulation, catecholamine release, endothelial dysfunction, oxidative stress, platelet activation, and increased blood viscosity [38,39]. Overall, smoking leads to both direct chemical effects and indirect consequences that promote CAS development.

While hypertension is more prevalent in classic angina than in angina induced by CAS [40], multiple studies indicate a link between low blood pressure and CAS. Specifically, low diastolic blood pressure (DBP) has been identified as a predictor of CAS [41,42,43]. This is further supported by our previous findings that non-hypertensive smokers face a greater CAS risk compared to their hypertensive counterparts, while hypertensive non-smoking women have the lowest risk [2]. In rats, the contractile response of rat coronary arteries to the CAS inducer, serotonin, diminishes with hypertension [44]. Furthermore, while synthetic phenotype smooth muscle cells are predominant in hypertensive rats, the contractile phenotype is primarily implicated in the pathogenesis of CAS [45]. Mechanistically, a decrease in blood pressure, particularly DBP, can reduce coronary perfusion, which may amplify the constrictor effects of endothelin, thereby contributing to CAS [46].

The heart’s endocrine function, confirmed in 1956, involves the natriuretic peptide system, which promotes significant natriuresis and diuresis, inhibits the renin–angiotensin–aldosterone axis, and acts as a vasodilator [47]. The possibility that myocardial ischemia may activate similar effector loops is a recent concept. While elevated BNP levels can indicate the severity of obstructive CAD [48], research shows that BNP levels are lower in acute coronary syndrome induced by CAS than in that induced by CAD [9], suggesting that BNP may help differentiate CAS-related angina in patients with non-obstructive CAD. BNP facilitates vasodilation by activating the natriuretic peptide receptor-A and increasing intracellular cGMP [49], opposing vasoconstrictive mechanisms such as the Renin–Angiotensin–Aldosterone System and the sympathetic nervous system. A deficiency in BNP can shift the balance toward vasoconstriction, potentially leading to CAS. Gaining a deeper understanding of BNP/natriuretic peptide receptor-A/cGMP signaling is crucial for comprehending receptor biology and CAS that result from abnormal hormone-receptor interactions within specific cells and tissues [49]. Consequently, an impaired BNP/natriuretic peptide receptor-A/cGMP signaling pathway cannot effectively induce vasorelaxation or oppose vasoconstrictors. In support of this, intravenous infusions of synthetic BNP, such as nesiritide [47], suppress hyperventilation-induced CAS [50], induce coronary vasodilation [47], and can completely reverse endothelin-mediated vasoconstriction [8]. These findings, combined with its longer half-life compared to atrial natriuretic peptide, position synthetic BNP as a promising future therapeutic option for CAS. Additionally, LDL cholesterol is a recognized risk factor for CAS, as oxidized LDL particles can impair endothelial function and reduce nitric oxide production [51].

Ischemia with no obstructive CAD (INOCA) is frequently underdiagnosed, as standard metrics such as global ejection fraction often appear normal despite ischemic symptoms. The condition involves complex pathophysiology, including the vasodilatory capacity of epicardial coronary vessels, which is regulated by both endothelial-dependent and -independent mechanisms. In a cohort of INOCA patients, the coronary lumen volume to myocardial mass ratio was significantly lower than in matched controls (25.6 ± 5.9 vs. 30.0 ± 6.5, *p* < 0.001) [52], emphasizing a potential link between epicardial vasodilatory capacity and LV myocardial mass. Furthermore, structural changes such as concentric remodeling, which causes LV diastolic dysfunction early in the ischemic cascade, are associated with a higher cardiovascular risk compared to normal geometry [53] and eccentric LV hypertrophy [54]. Findings from the WISE study corroborate this, showing that women with intermediate coronary flow reserve and lower myocardial perfusion reserve were more likely to have concentric remodeling and impaired LV diastolic function compared to those with higher myocardial perfusion reserve, providing evidence that both coronary vasomotion and concentric remodeling are significant contributors to myocardial ischemia [55,56]. In INOCA, a cycle of impaired vasomotion, concentric remodeling, and fibrosis can reduce coronary flow reserve and increase metabolic demand [56]. Our multivariable analysis revealed that in patients with CAS, RWTLVESD was a more significant risk factor than RWTLVEDD, suggesting that CAS, along with impaired relaxation, can reduce LV compliance and increase filling pressure. Regarding the relation to concentric remodeling in our CAS patients, mitral lateral annular e’ velocity demonstrated negative correlations with RWTLVEDD (r = −0.199, *p* < 0.001), RWTLVESD (r = −0.152, *p* < 0.001) and Tei index (r = −0.316, *p* < 0.001), while showing a positive correlation with E-wave velocity (r = 0.330, *p* < 0.001). Although our CAS and control patients had significantly different LVMIs, they were within normal range; hence, in our CAS patients, who largely had normal LVMI, impairments in relaxation appeared to be a direct cause of diastolic dysfunction. Notably, in the WISE study, LV diastolic echocardiographic parameters, such as mitral annular velocities and Tei index, were not investigated, limiting their applicability [55,56]. Women with INOCA showed lower diastolic strain rates and ventricular untwisting (measured by early Doppler myocardial tissue velocities and IVRT) compared to controls, leading to reduced e’(l), e’(m), and prolonged IVRT, consistent with our results. This is also in accordance with previous findings demonstrating that a reduced diastolic function, as determined by a reduced early diastolic relaxation tissue velocity e′, is a strong predictor of acute coronary syndrome [57]. We demonstrated for the first time an association between CAS and concentric remodeling, which may lead to myocardial capillary rarefaction and worsen diastolic function. This suggests a potential positive feedback loop between CAS, diastolic dysfunction, and concentric remodeling.

The Tei index, a reliable and reproducible measure of global myocardial performance, combines systolic and diastolic time intervals and is notably independent of heart rate and loading conditions. This independence makes it particularly useful for evaluating remodeled hearts, where altered preload and afterload can interfere with standard load-dependent echocardiographic parameters such as ejection fraction. For instance, in cardio-oncology, a worsening Tei index has been identified as a sensitive indicator of silent cardiotoxicity from 5-fluorouracil, a drug known to induce CAS [58]. The index shows a strong correlation with invasive measurements of cardiac function. It is inversely related to cardiac output and directly correlated with metrics such as systolic and diastolic peak dP/dT, ventricular stiffness, and tau in patients with ischemic heart disease [59]. We further demonstrated that in CAS, the Tei index was negatively related to mitral E-wave velocity (r = −0.388, *p* < 0.001) and BNP (r = −0.128, *p* = 0.001), while positively correlating with DBP (r = 0.103, *p* = 0.009), τ_0l_ (r = 0.208, *p* < 0.001) and τ_0m_ (r = 0.202, *p* < 0.001). These established correlations underscore its reliability for evaluating left ventricular function in the context of CAS.

**Figure 3:**
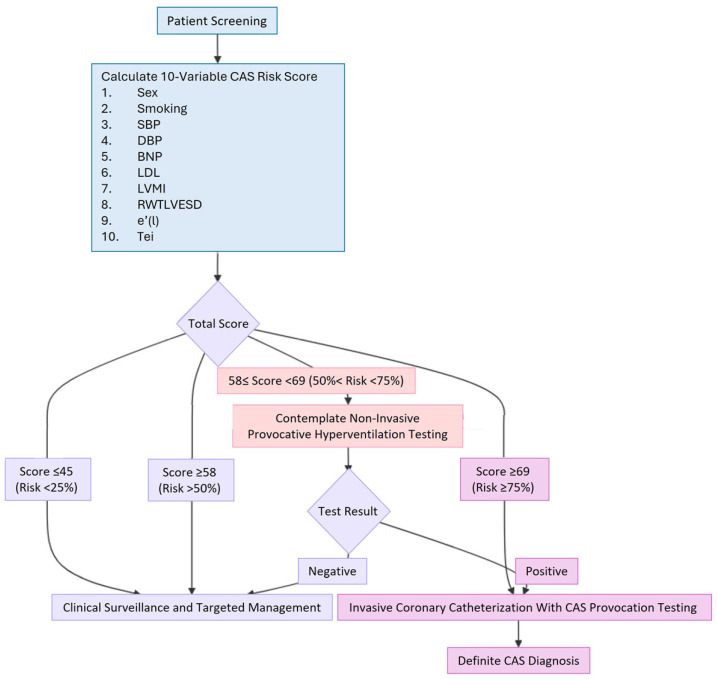
A concise flowchart diagram of the clinical pathway. BNP: B-type natriuretic peptide; CAS: coronary artery spasm; DBP: diastolic blood pressure; e’(l): tissue Doppler-derived early diastolic velocities from the lateral mitral annulus; LDL: low-density lipoprotein; LVMI: left ventricular mass index; RWTLVESD: relative wall thickness using LVESD; SBP: systolic blood pressure.

The 10-variable CAS risk score facilitates a tiered clinical pathway for patient diagnosis and management. The process begins by calculating easily accessible clinical information, blood test results, and echocardiographic measurements—including sex, smoking status, SBP, DBP, BNP, LDL, LVMI, RWTLVESD, (e’(l)), and Tei index—to enable rapid, non-invasive risk stratification. A score of ≤45 indicates a low probability of CAS (≤25%), for which clinical follow-up and targeted testing based on the patient’s evolving condition are recommended. For patients with a score of ≥58 (predicted risk >50%), an intermediate likelihood of CAS is indicated, and non-invasive provocative hyperventilation testing should be contemplated. Finally, a high score of ≥69 (probability ≥75%) strongly recommends invasive coronary catheterization with CAS provocation testing to achieve a definite diagnosis [60] (Figure 3).

### Study Limitations

First, the study subjects were patients from Shuang Ho Hospital, a reference center known for the quality of its interventional care, particularly in coronary angiography with CAS provocation testing and myocardial revascularization. This could be a source of selection bias, but analysis of the data used to validate the risk scores revealed that the study sample included the full spectrum of CAS. Second, any risk estimation systems will underestimate risk if cardiovascular events have increased. Therefore, recalibration should be undertaken if good-quality prevalence data on contemporary events and risk factors are available. Third, the findings of this study were derived from a single-center cohort composed of an ethnically homogeneous Taiwanese population. As a result, the applicability of the model to other ethnic groups or healthcare settings may be limited. Differences in genetic backgrounds, disease epidemiology, clinical practice patterns, and healthcare systems across regions may influence model performance. Therefore, external validation in independent cohorts from diverse populations is essential to confirm the model’s generalizability and ensure its broader clinical utility. Fouth, our goal was to use common echocardiographic parameters and blood test variables to build a predictive model for CAS. It is important to note that two biomarkers, BNP and LDL, identified in our CAS patients were nonspecific and recognized as established biomarkers/risk factors for obstructive CAD. This highlights the complexity of CAS, where multiple pathological processes occur at once, making it difficult to identify a single promising circulating biomarker. Although the biomarkers we studied are easily accessible and useful in clinical practice, BNP and LDL are nonspecific for CAS. Hence, the inclusion of some specific biomarkers, beyond BNP, could be of great help for CAS prediction [61]. Given these limitations, several biomarkers show potential in CAS but require validation in larger, mechanistic studies with objective CAS diagnosis. Fifth, although the 2010 UK NICE guidelines recommended cardiac CT angiography (CTCA) as the initial investigation for patients presenting with new-onset angina associated with obstructive CAD [62], several concerns have since emerged. Increased therapy for risk factor modification has contributed to an overestimation of significant CAD prevalence, particularly among women. The use of coronary artery calcium score as the primary investigation, followed by CTCA, has also been questioned; the UK Royal College of Radiologists indicated that CTCA is appropriate for individuals with a calcium score of 0. Even in 2010, CTCA was shown to benefit patients with a low or intermediate pre-test likelihood of CAD. Consequently, according to the 2016 NICE guideline [63], CTCA is no longer recommended as the first-line test for every patient with atypical or typical angina. Consequently, if CTCA reveals normal epicardial coronary arteries in a patient, the likelihood of CAS increases. Sixth, this study aimed to assess how well routine echocardiographic parameters predict CAS, not to introduce new measurement methods. While all the echocardiographic measurements were made by a blinded observer within a core lab setting to ensure internal validity and reliability, our study did not involve different observers taking the same measurements in a patient and/or an observer re-evaluating the same image at different times. Since one physician performed all echocardiographic assessments using consistent techniques and reliable tools, measurement variability should have been minimal [64]. Conversely, the lack of data regarding interobserver variability constituted a significant limitation. Further investigation is recommended. Seventh, both the RWTLVESD and Tei index are not inherently machine- or vendor-specific, but rather are standardized calculations based on fundamental echocardiographic measurements in echocardiography guidelines. However, inter-vendor differences in results may arise from variations in ultrasound technology or image processing, though the echocardiographic parameters used are standard and not exclusive to any manufacturer. Therefore, to ensure clinical consistency, feasibility and reproducibility of measuring these indices in other institutions with different equipment, it is advisable to use the same echocardiography machine and software settings. Furthermore, it is acknowledged that clinical practice patterns may have undergone some refinement and evolution over the extended data collection period (2008–2025), which could influence the generalizability of our findings. Lastly, because one clinician managed all patients, operator-related bias may affect these findings.

## 5. Conclusions

Our study presents a simple, comprehensive 10-variable scoring model that combines clinical, echocardiographic, and blood test data—including sex, smoking status, SBP, DBP, BNP, LDL, LVMI, RWTLVESD, e’(l), and Tei index—to non-invasively identify patients at risk of developing CAS. The CAS risk score provides a clear framework for clinical action: a score of ≤45 (low probability ≤ 25%) suggests clinical follow-up with testing tailored to the patient’s evolving condition; a score of ≥58 (intermediate probability > 50%) indicates that non-invasive provocative hyperventilation testing should be considered; and a score of ≥69 (high probability ≥ 75%) strongly recommends invasive coronary catheterization with provocation testing for a definitive diagnosis. LV concentric remodeling and diastolic dysfunction may contribute to CAS, warranting future investigation of specific mechanisms and treatment options. Our well-calibrated CAS risk score model serves as a screening tool with good discrimination, enabling efficient allocation of diagnostic coronary catheterization, individualized treatment, and improved outcomes.

## Figures and Tables

**Table 1:** Baseline characteristics of patients in the CAS and non-CAS control groups.

Variable	Total (*n* = 913)	CAS (*n* = 645)	Control (*n* = 268)	*p* Value
Age, year	56.4 ± 12.6	57.1 ± 11.9	54.8 ± 14.2	0.014
Male	453 (49.6)	352 (54.6)	101 (37.7)	<0.001
Body mass index, kg/m^2^	26.1 ± 4.4	26.2 ± 4.2	25.8 ± 4.6	0.187
Weight status				0.097
Normal/Lean (<23)	226 (24.8)	147 (22.8)	79 (29.5)	
Overweight (23–24.9)	174 (19.1)	124 (19.2)	50 (18.7)	
Obese (≥25)	513 (56.2)	374 (58.0)	139 (51.9)	
Body surface area, m^2^	1.76 ± 0.20	1.77 ± 0.20	1.73 ± 0.21	0.009
Smoke	229 (25.1)	184 (28.5)	45 (16.8)	<0.001
Diabetes mellitus	109 (11.9)	81 (12.6)	28 (10.4)	0.433
Hypertension	301 (33.0)	220 (34.1)	81 (30.2)	0.279
Systolic blood pressure, mmHg	122.0 ± 18.9	120.2 ± 17.7	126.3 ± 20.9	<0.001
Diastolic blood pressure, mmHg	74.3 ± 10.6	73.4 ± 10.3	76.6 ± 11.0	<0.001
Heart rate, bpm	69.1 ± 11.5	68.7 ± 11.7	70.2 ± 11.1	0.072
Serum creatinine, mg/dL	0.86 ± 0.31	0.85 ± 0.27	0.87 ± 0.39	0.520
Estimated glomerular filtration rate, mL/min/1.73 m^2^	91.6 ± 23.1	92.2 ± 22.0	90.1 ± 25.6	0.227
Hemoglobin, g/dL	13.6 ± 1.5	13.8 ± 1.5	13.3 ± 1.5	<0.001
Hematocrit, %	40.1 ± 4.4	40.6 ± 4.2	39.0 ± 4.5	<0.001
Platelet counts, 10^9^/L	231.4 ± 58.7	232.0 ± 59.4	229.9 ± 57.1	0.618
White blood cell count, 10^6^/L	6822.6 ± 1730.9	6874.0 ± 1790.0	6698.8 ± 1576.1	0.164
Monocyte counts, 10^6^/L	495.2 ± 165.2	501.9 ± 168.4	479.2 ± 156.5	0.058
B-type natriuretic peptide, pg/mL	20.9 [9.0, 42.8]	20.3 [7.6, 40.0]	23.0 [10.3, 48.5]	0.078
High-sensitivity C-reactive protein, mg/L	0.82 [0.40, 1.91]	0.90 [0.40, 2.01]	0.70 [0.39, 1.64]	0.074
High-sensitivity C-reactive protein, mg/L				0.112
≤1	505 (55.3)	344 (53.3)	161 (60.1)	
1–2.9	266 (29.1)	192 (29.8)	74 (27.6)	
≥3	142 (15.6)	109 (16.9)	33 (12.3)	
Fasting glucose, mg/dL	100.3 ± 19.5	101.0 ± 19.7	98.8 ± 18.8	0.118
Glycated hemoglobin, %	5.76 ± 0.66	5.79 ± 0.68	5.71 ± 0.61	0.112
Total cholesterol, mg/dL	170.6 ± 36.4	171.7 ± 36.5	168.0 ± 35.9	0.160
Triglycerides, mg/dL	122.4 ± 83.9	126.8 ± 86.9	112.0 ± 75.3	0.015
High-density lipoprotein, mg/dL	46.2 ± 12.8	45.6 ± 12.4	47.6 ± 13.7	0.033
Low-density lipoprotein, mg/dL	100.4 ± 31.2	101.6 ± 31.0	97.6 ± 31.4	0.075
Echocardiography				
LV ejection fraction, %	65.4 ± 6.9	65.6 ± 6.8	64.9 ± 7.3	0.154
Fractional shortening	0.36 ± 0.06	0.36 ± 0.06	0.36 ± 0.06	0.403
Left atrium diameter, mm	38.6 ± 6.1	38.8 ± 6.0	38.2 ± 6.2	0.159
Interventricular septal thickness, mm	9.18 ± 1.81	9.28 ± 1.74	8.95 ± 1.96	0.014
LVEDD, mm	44.4 ± 4.7	44.6 ± 4.6	44.1 ± 4.7	0.139
LVESD, mm	28.4 ± 4.2	28.4 ± 4.2	28.2 ± 4.1	0.406
Left ventricular posterior wall, mm	8.41 ± 1.55	8.63 ± 1.44	7.89 ± 1.66	<0.001
RWTLVEDD	0.38 ± 0.08	0.39 ± 0.08	0.36 ± 0.08	<0.001
RWTLVESD	0.60 ± 0.14	0.62 ± 0.13	0.57 ± 0.14	<0.001
LV mass, g	131.3 ± 39.5	134.8 ± 37.9	122.8 ± 42.0	<0.001
LV mass index, g/m^2^	74.3 ± 19.4	76.0 ± 18.6	70.4 ± 20.9	<0.001
LV remodeling				0.012
Normal	633 (69.3)	429 (66.5)	204 (76.1)	
Concentric remodeling	239 (26.2)	187 (29.0)	52 (19.4)	
Concentric hypertrophy	26 (2.8)	20 (3.1)	6 (2.2)	
Eccentric hypertrophy	15 (1.6)	9 (1.4)	6 (2.2)	
Mitral inflow deceleration time, ms	188.7 ± 34.6	189.9 ± 34.5	185.8 ± 34.9	0.100
Isovolumic relaxation time, ms	86.5 ± 12.6	87.3 ± 12.5	84.6 ± 12.7	0.003
τ_0_l: LV relaxation time constant using lateral annulus, ms	37.8 ± 6.8	38.4 ± 6.6	36.4 ± 7.1	<0.001
τ_0_m: LV relaxation time constant using medial annulus, ms	40.4 ± 7.6	40.9 ± 7.3	39.3 ± 8.1	0.004
E: Mitral inflow E-wave velocity, cm/s	60.8 ± 15.5	59.5 ± 15.2	64.0 ± 15.7	<0.001
A: Mitral inflow A-wave velocity, cm/s	59.1 ± 16.9	59.0 ± 16.2	59.3 ± 18.5	0.828
E/A: Mitral inflow E/A ratio	1.11 ± 0.44	1.08 ± 0.41	1.19 ± 0.51	0.001
e′(l): Lateral mitral annular early diastolic velocity, cm/s	7.95 ± 2.54	7.73 ± 2.41	8.47 ± 2.77	<0.001
a′(l): Lateral mitral annular late diastolic velocity, cm/s	9.36 ± 2.26	9.28 ± 2.21	9.55 ± 2.37	0.103
e′(m): Medial mitral annular early diastolic velocity, cm/s	6.38 ± 1.93	6.27 ± 1.86	6.63 ± 2.08	0.010
a′(m): Medial mitral annular late diastolic velocity, cm/s	8.76 ± 1.72	8.76 ± 1.69	8.74 ± 1.77	0.863
E/e′(l): Lateral mitral inflow E to e′ ratio	8.25 ± 2.95	8.27 ± 2.89	8.20 ± 3.07	0.760
E/e′(m): Medial mitral inflow E to e′ ratio	10.20 ± 3.46	10.09 ± 3.28	10.46 ± 3.87	0.146
Tei index	0.48 ± 0.16	0.50 ± 0.15	0.45 ± 0.15	<0.001

CAS = coronary artery spasm; LV = left ventricle; LVEDD = Left ventricular end-diastolic diameter; LVESD = Left ventricular end-systolic diameter; RWTLVEDD = Relative wall thickness using LVEDD; RWTLVESD = Relative wall thickness using LVESD. Data are presented as frequency (percentage), mean ± standard deviation or median [25th percentile, 75th percentile].

**Table 2:** Univariate logistic regression analysis of the potential association between the baseline characteristics and risk of coronary artery spasm.

Predictor	Odds Ratio (95% CI)	*p* Value
Age, per 10 years	1.01 (1.003–1.03)	0.014
Male sex	1.99 (1.48–2.66)	<0.001
Body mass index, per 5 kg/m^2^	1.12 (0.95–1.32)	0.187
Weight status		
Normal/Lean (<23 kg/m^2^)	Reference	–
Overweight (23–24.9 kg/m^2^)	1.33 (0.87–2.04)	0.188
Obese (≥25 kg/m^2^)	1.45 (1.03–2.02)	0.031
Body surface area, per 0.1 m^2^	1.10 (1.02–1.18)	0.009
Smoke	1.98 (1.38–2.84)	<0.001
Diabetes mellitus	1.23 (0.78–1.94)	0.371
Hypertension	1.20 (0.88–1.63)	0.256
Systolic blood pressure, per 10 mmHg	0.85 (0.78–0.91)	<0.001
Diastolic blood pressure, per 10 mmHg	0.75 (0.65–0.86)	<0.001
Heart rate, per 5 bpm	0.95 (0.89–1.01)	0.073
Serum creatinine, per 0.1 mg/dL	0.99 (0.94–1.03)	0.521
eGFR, per 10 mL/min/1.73 m^2^	1.04 (0.98–1.11)	0.227
Hemoglobin, per 1 g/dL	1.23 (1.12–1.36)	<0.001
Hematocrit, per 5%	1.50 (1.27–1.77)	<0.001
Platelets, per 10^12^/L	1.001 (0.998–1.003)	0.618
WBC, per 10^9^/L	1.06 (0.98–1.15)	0.164
Monocytes, per 10^9^/L	1.09 (0.997–1.19)	0.059
B-type natriuretic peptide, per 1 log unit	0.97 (0.95–0.999)	0.041
hs-CRP, mg/L	1.003 (0.99–1.02)	0.765
High-sensitivity C-reactive protein, mg/L		
≤1	Reference	–
1–2.9	1.21 (0.88–1.68)	0.245
≥3	1.55 (1.003–2.38)	0.048
Fasting glucose, per 10 mg/dL	1.06 (0.98–1.15)	0.120
Glycated hemoglobin, per 0.1%	1.02 (0.996–1.04)	0.113
Total cholesterol, per 10 mg/dL	1.03 (0.99–1.07)	0.160
Triglycerides, per 10 mg/dL	1.02 (1.004–1.05)	0.016
High-density lipoprotein, per 10 mg/dL	0.89 (0.80–0.99)	0.034
Low-density lipoprotein, per 10 mg/dL	1.04 (0.996–1.09)	0.076
LV ejection fraction, per 5%	1.08 (0.97–1.19)	0.154
Fractional shortening, per 10%	1.10 (0.88–1.38)	0.402
Left atrium diameter, per 5 mm	1.09 (0.97–1.22)	0.159
Interventricular septal thickness, per 1 mm	1.11 (1.02–1.20)	0.014
LVEDD, per 1 mm	1.02 (0.99–1.06)	0.139
LVESD, per 1 mm	1.02 (0.98–1.05)	0.406
Left ventricular posterior wall, per 1 mm	1.41 (1.27–1.57)	<0.001
RWTLVEDD, per 0.1	1.66 (1.37–2.03)	<0.001
RWTLVESD, per 0.1	1.34 (1.19–1.50)	<0.001
LV mass, per 10 g	1.09 (1.05–1.13)	<0.001
LV mass index, per 10 g/m^2^	1.18 (1.08–1.27)	<0.001
LV remodeling		
Normal	ref	ref
Concentric remodeling	1.71 (1.21–2.43)	0.003
Concentric hypertrophy	1.59 (0.63–4.01)	0.330
Eccentric hypertrophy	0.71 (0.25–2.03)	0.527
Mitral inflow deceleration time, per 10 ms	1.04 (0.99–1.08)	0.100
Isovolumic relaxation time, per 10 ms	1.19 (1.06–1.34)	0.003
τ_0_l, per 10 ms	1.55 (1.25–1.94)	<0.001
τ_0_m, per 10 ms	1.33 (1.09–1.62)	0.005
E, per 10 m/s	0.83 (0.76–0.91)	<0.001
A, per 10 m/s	0.99 (0.91–1.08)	0.828
E/A, per 0.1	0.95 (0.92–0.98)	0.001
e′(l), per 0.1 m/s	0.99 (0.98–0.99)	<0.001
a′(l), per 0.1 m/s	0.995 (0.99–1.001)	0.103
e′(m), per 0.1 m/s	0.99 (0.98–0.998)	0.011
a′(m), per 0.1 m/s	1.001 (0.99–1.01)	0.863
E/e′(l), per 0.1	1.001 (0.996–1.01)	0.761
E/e′(m), per 0.1	0.997 (0.99–1.001)	0.146
Tei index, per 0.1	1.22 (1.11–1.34)	<0.001

CI = confidence interval; eGFR = estimated glomerular filtration rate; WBC = white blood count; hs-CRP = high-sensitivity C-reactive protein; LV = left ventricle; LVEDD = left ventricular end-diastolic diameter; LVESD = left ventricular end-systolic diameter; RWTLVEDD = relative wall thickness using LVEDD; RWTLVESD = relative wall thickness using LVESD.

**Table 3:** Multivariable logistic regression analysis of factors associated with a higher risk of coronary artery spasm.

Predictor	Estimate	Odds Ratio (95% CI)	*p* Value
Intercept	2.13	-	-
Male sex	0.57	1.76 (1.26–2.47)	0.001
Smoke	0.56	1.74 (1.16–2.62)	0.008
Systolic blood pressure, per 10 mmHg	−0.15	0.86 (0.78–0.95)	0.002
Diastolic blood pressure, per 10 mmHg	−0.36	0.70 (0.59–0.84)	<0.001
B-type natriuretic peptide, per 10 pg/mL	−0.04	0.96 (0.93–0.99)	0.016
Low-density lipoprotein, per 10 mg/dL	0.05	1.05 (1.001–1.11)	0.046
RWTLVESD, per 0.1	0.25	1.29 (1.14–1.46)	<0.001
Left ventricular mass index, per 10 g/m^2^	0.16	1.17 (1.06–1.29)	0.002
e′(l), per 0.1 m/s	−0.01	0.99 (0.98–0.996)	0.003
Tei index, per 0.1	0.17	1.18 (1.06–1.32)	0.003

CI = confidence interval; RWTLVESD = relative wall thickness at left ventricular end-systole.

**Table 4:** Simplified score function of the prediction model for coronary artery spasm.

Point Values for Each Variable				Summation of Points	
Variable	Point	Variable	Point	Total Points	Probability of CAS
Sex		RWTLVESD		36	0.12
Female	0	<0.35	0	37	0.13
Male	6	0.35–0.39	1	38	0.14
Smoke		0.40–0.44	3	39	0.15
No	0	0.45–0.49	4	40	0.16
Yes	6	0.50–0.54	5	41	0.18
SBP, mmHg		0.55–0.59	7	42	0.19
<100	15	0.60–0.64	8	43	0.21
100–109	13	0.65–0.69	9	44	0.22
110–119	11	0.70–0.74	11	45	0.24
120–129	10	0.75–0.79	12	46	0.26
130–139	8	0.80–0.84	13	47	0.27
140–149	7	0.85–0.89	15	48	0.29
150–159	5	0.90–0.94	16	49	0.31
160–169	3	0.95–0.99	17	50	0.33
170–179	2	1–1.04	19	51	0.35
≥180	0	≥1.05	20	52	0.38
DBP, mmHg		LVMI, g/m^2^		53	0.40
<55	19	<50	0	54	0.42
55–59	17	50–59	2	55	0.44
60–64	15	60–69	3	56	0.47
65–69	13	70–79	5	57	0.49
70–74	11	80–89	7	58	0.51
75–79	9	90–99	8	59	0.54
80–84	8	100–109	10	60	0.56
85–89	6	110–119	12	61	0.58
90–94	4	120–129	13	62	0.61
95–99	2	130–139	15	63	0.63
≥100	0	140–149	17	64	0.65
BNP, pg/mL		≥150	18	65	0.67
<50	13	e′(l), cm/s		66	0.69
50–99	11	3	13	67	0.71
100–149	9	4	12	68	0.73
150–199	7	5	11	69	0.75
200–249	4	6	10	70	0.77
250–299	2	7	9	71	0.78
≥300	0	8	8	72	0.80
LDL, mg/dL		9	7	73	0.81
<30	0	10	6	74	0.83
31–40	1	11	4	75	0.84
41–60	2	12	3	76	0.85
61–80	3	13	2	77	0.87
81–100	4	14	1	78	0.88
101–120	6	15	0	79	0.89
121–140	7	Tei index		80	0.90
141–160	8	<0.2	0	82	0.91
161–180	9	0.2	2	83	0.92
≥180	10	0.3	4	85	0.93
		0.4	5	86	0.94
		0.5	7	88	0.95
		0.6	9	91	0.96
		0.7	11	94	0.97
		0.8	12	98	0.98
		>0.8	14		

BNP = B-type natriuretic peptide; DBP = diastolic blood pressure; LDL = low-density lipoprotein; LVMI = left ventricular mass index; RWTLVESD = relative wall thickness at left ventricular end-systole; SBP = systolic blood pressure.

## Data Availability

The data presented in this study are available upon request from the corresponding author.

## Abbreviations

BNP: B-type natriuretic peptide; CAD: coronary artery disease; CAS: coronary artery spasm; cGMP: cyclic guanosine monophosphate; CTCA: cardiac CT angiography; DBP: diastolic blood pressure; e’(l): tissue Doppler-derived early diastolic velocities from the lateral mitral annulus; INOCA: ischemia with non-obstructive coronary artery disease; IVRT: isovolumic relaxation time; LDL: low-density lipoprotein; LVMI: left ventricular mass index; RWT: relative wall thickness; RWTLVEDD: relative wall thickness at end-diastole; RWTLVESD: relative wall thickness at end-systole; SBP: systolic blood pressure.
